# *The Organon* and Materia Medica Club of the Bay Cities of California

**Published:** 1898-06

**Authors:** Guy E. Manning

**Affiliations:** Secretary


### *THE ORGANON* AND MATERIA MEDICA CLUB OF THE BAY CITIES OF CALIFORNIA.

The *Organon* and Materia Medica Club was called to order by President J. M. Selfridge December 3d, 1897, at 8.30 P. M.

Those present were Drs. J. M. Selfridge, Holmgren, Augur, and Manning.

The reading of *The Organon*, Sections 21 to 28, was continued.

Sec. 21 was stated to be a pleading for proving of drugs.

Section 22:

Dr. Augur—I do not understand his meaning a patient is not conscious of drug disease.

Dr. Selfridge—The line of argument is you cannot know what a drug will do or what produce until it has been proved. Proving is the only way of finding out.

Dr. Augur—I do not know how he can use the term "artificial symptoms" when there are none in a proving.

Dr. Selfridge—Perhaps it is a wrong term, but if we go back to his term "disease force," then we can see his point.

Section 24:

Dr. Manning—Do you consider that a cure is ever performed except homœopathically?

Dr. Selfridge—No; there may be a palliation, but not a cure.

Dr. Manning—Then no cure takes place unless the proper remedy has been given.

Dr. Selfridge—Cures exist from no medicine, but the proper remedy will always cure.

Discussion then ended, followed by a paper "A Case of Jaundice," by Dr. Selfridge.

Adjourned on motion.

GUY E. MANNING,
*Secretary.*

